# Supplementary material for: Multi‐dataset identification of innovative feature genes and molecular mechanisms in keratoconus
Source: J Cell Mol Med. 2024 Sep 19;28(18):e70079. doi: 10.1111/jcmm.70079 (PMC11412914; doi:10.1111/jcmm.70079)
Supplement: Supplementary file 2 — Material S2. [file JCMM-28-e70079-s002.zip › 7.GSEA analysis/2.ATOH7/KEGG_PRIMARY_BILE_ACID_BIOSYNTHESIS.html]

Details for gene set KEGG\_PRIMARY\_BILE\_ACID\_BIOSYNTHESIS[GSEA]

|  || Dataset | ATOH7.group.cls#h\_versus\_l |
| Phenotype | group.cls#h\_versus\_l |
| Upregulated in class | h |
| GeneSet | KEGG\_PRIMARY\_BILE\_ACID\_BIOSYNTHESIS |
| Enrichment Score (ES) | 0.65454227 |
| Normalized Enrichment Score (NES) | 1.4687423 |
| Nominal p-value | 0.046153847 |
| FDR q-value | 0.26872903 |
| FWER p-Value | 0.822 |
Table: GSEA Results Summary

  

Fig 1: Enrichment plot: KEGG\_PRIMARY\_BILE\_ACID\_BIOSYNTHESIS      
 Profile of the Running ES Score & Positions of GeneSet Members on the Rank Ordered List

  

| SYMBOL | TITLE | RANK IN GENE LIST | RANK METRIC SCORE | RUNNING ES | CORE ENRICHMENT || 1 | CH25H | na | 240 | 0.650 | 0.1735 | Yes |
| 2 | CYP27A1 | na | 577 | 0.525 | 0.3094 | Yes |
| 3 | AKR1C4 | na | 843 | 0.472 | 0.4327 | Yes |
| 4 | ACOX2 | na | 2297 | 0.323 | 0.4805 | Yes |
| 5 | CYP46A1 | na | 4361 | 0.223 | 0.4830 | Yes |
| 6 | AMACR | na | 5154 | 0.196 | 0.5146 | Yes |
| 7 | CYP39A1 | na | 5185 | 0.195 | 0.5679 | Yes |
| 8 | CYP7B1 | na | 5469 | 0.186 | 0.6115 | Yes |
| 9 | AKR1D1 | na | 5702 | 0.179 | 0.6545 | Yes |
| 10 | CYP8B1 | na | 7457 | 0.132 | 0.6405 | No |
| 11 | HSD17B4 | na | 14531 | 0.025 | 0.4439 | No |
| 12 | SLC27A5 | na | 20617 | -0.028 | 0.2763 | No |
| 13 | SCP2 | na | 21019 | -0.032 | 0.2736 | No |
| 14 | HSD3B7 | na | 24905 | -0.084 | 0.1849 | No |
| 15 | BAAT | na | 25243 | -0.090 | 0.2001 | No |
| 16 | CYP7A1 | na | 31395 | -0.263 | 0.0960 | No |
Table: GSEA details [plain text format]

  

Fig 2: KEGG\_PRIMARY\_BILE\_ACID\_BIOSYNTHESIS      
 Blue-Pink O' Gram in the Space of the Analyzed GeneSet

  

Fig 3: KEGG\_PRIMARY\_BILE\_ACID\_BIOSYNTHESIS: Random ES distribution      
 Gene set null distribution of ES for **KEGG\_PRIMARY\_BILE\_ACID\_BIOSYNTHESIS**
